# *Pseudomonas aeruginosa* elastase down-regulates host inflammatory responses by degrading cytokines and chemokines: a non-healing wound perspective

**DOI:** 10.3389/fmed.2025.1585252

**Published:** 2025-06-24

**Authors:** Mariena J. A. van der Plas, Manoj Puthia, Seow Theng Ong, Kajsa Arkelius, Ann-Charlotte Strömdahl, Marta Butrym, Magnus Rasmussen, Navin Kumar Verma, Artur Schmidtchen

**Affiliations:** ^1^Division of Dermatology and Venereology, Department of Clinical Sciences, Lund University, Lund, Sweden; ^2^LEO Foundation Center for Cutaneous Drug Delivery, Department of Pharmacy, University of Copenhagen, Copenhagen Ø, Denmark; ^3^Lee Kong Chian School of Medicine, Nanyang Technological University, Singapore, Singapore; ^4^Division of Infection Medicine, Department of Clinical Sciences, Lund University, Lund, Sweden; ^5^Division of Infectious Diseases, Skane University Hospital, Lund, Sweden; ^6^Department of Dermatology, Skane University Hospital, Lund, Sweden

**Keywords:** wound infection, porcine (pig) model, proteolytic acitivity, LasB elastase, monocytes, blood, clinical isolates

## Abstract

Non-healing venous leg ulcers are characterized by dysfunctional wound healing and frequently exhibit an absence of classical inflammatory signs, despite substantial bacterial loads of the Gram-negative pathogen *Pseudomonas aeruginosa*. To investigate this clinical observation, we used a porcine wound infection model and complementary *in vitro* cell and enzymatic activity assays. *In vivo*, *P. aeruginosa* infected wounds resulted in attenuated inflammatory responses compared to those infected with *Staphylococcus aureus*. Protease activity was elevated in *P. aeruginosa*-infected wounds relative to uninfected controls, while pro-inflammatory cytokine levels decreased over time. *In vitro* analyses employing cell cultures, wildtype and mutant strains, and clinical isolates from venous ulcers and blood, revealed that *P. aeruginosa* elastase (LasB) degrades a range of pro-inflammatory cytokines (G-CSF, GM-CSF, IFN-γ, IL-1ra, IL-6, IL-12p40, IL-23, TNF-α) and chemokines (Gro-α, IL-8, IP-10, MCP-1, MIP-1α, MIP-1β) in the extracellular milieu, without impacting cell morphology, transcription factor activation, or subsequent intracellular cytokine production. Correspondingly, wound fluids from non-healing ulcers colonized/infected with *P. aeruginosa* degraded cytokines, whereas fluids from uninfected wounds did not. Collectively, our findings indicate that *P. aeruginosa* modulates host inflammation by degrading cytokines and chemokines.

## Introduction

*Pseudomonas aeruginosa* is a Gram-negative opportunistic pathogen leading to substantial morbidity and mortality in immuno-compromised and critically ill patients as well as individuals with for example cystic fibrosis, burns and non-healing wounds (1–3). The significance of disease caused by *P. aeruginosa* is underscored by WHO, as it placed these bacteria on the latest (2024) priority list of pathogens with critical need for new treatments due to increasing antibiotic resistance.

Over the past few decades, numerous studies have identified the virulence factors, particularly those regulated by the quorum sensing *lasR* gene complex and dependent on the Type II (T2SS) and Type III (T3SS) secretion systems, enabling *P. aeruginosa* to manipulate host immunity, survive at infection sites, and disseminate [see detailed reviews here (4–9)]. For example, lipopolysaccharides, flagellin, and the Large exoprotease (lepA) (10) increase activation of signal transcription factor NF-κB and its downstream gene expression, Exotoxin A and alginate increase reactive oxygen species (ROS) and cytokine production by monocytes (11–13), lipase (14) and ExoS (15) may decrease ROS levels, whereas LecB can either induce or inhibit intracellular ROS production in neutrophils depending on the circumstances (16). Furthermore, it has been shown that *P. aeruginosa* proteases, including elastase A (LasA), elastase B (LasB), alkaline protease (AprA), and protease IV (PIV) can degrade a variety of host proteins [reviewed here (6, 17)], including components of the extracellular matrix (18–22), cytokines and chemokines (23–26), the complement system (22, 27, 28), immunoglobulins (22, 27, 29), and antimicrobial peptides (27, 30). Recently, we used a peptidomics approach to extensively map the activity of the metalloprotease elastase B (LasB, pseudolysin) in human plasma and wound fluids leading to the identification of respectively 145 and 155 unique protein targets in these fluids, which included a range of serum proteins, complement factors, apolipoproteins, proteases and their inhibitors, histones, as well as clot and extracellular matrix components (27). Moreover, at a global level, *P. aeruginosa* infected porcine and human wounds showed a unique peptidome and protease signature (31). Although protein degradation often leads to loss of function, in some cases the resulting peptides exert novel actions unrelated to the parent protein. Indeed, we discovered that elastase degrades thrombin, thereby releasing a peptide that inhibits inflammatory responses by preventing toll-like receptor (TLR) dimerization, and which was identified in non-healing *P. aeruginosa* infected wounds (32).

**FIGURE 1:**
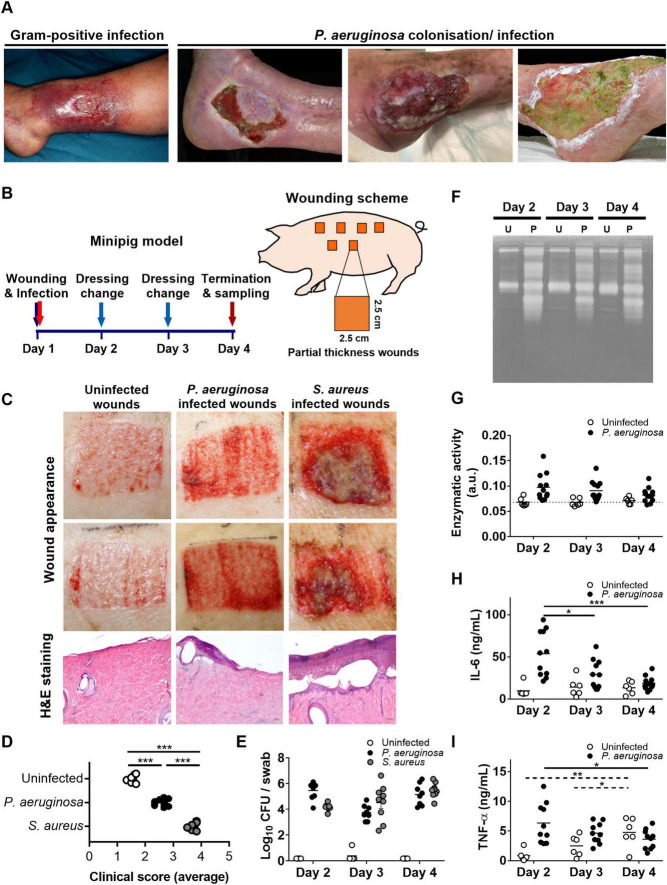
Comparison of *P. aeruginosa* infections and Gram-positive infections in wounds. **(A)** Representative images of wounds infected with Gram-positive bacteria or Gram-negative *P. aeruginosa*. **(B)** Schematic overview of the porcine wound model. Partial thickness wounds of 6.25 cm^2^, 750 μm deep created on the dorsal side of Göttingen minipigs, were infected with 10^7^ CFUs of *P. aeruginosa* PAO1 or *S. aureus* ATCC 29213 followed by daily dressing changes for 2 days and finally termination of the experiment at day 4. **(C)** Wound appearance and histology of biopsies at day 4 show moderate inflammation and cell infiltration and reduced re-epithelialization for *P. aeruginosa* infected wounds as compared to uninfected wounds. Contrastingly, *S. aureus* infected wounds show extensive inflammation, cell infiltration and tissue necrosis. **(D)** Wounds at day 4 were scored based on 6 parameters (dressing stickiness, exudate amount, exudate type, presence of necrotic tissue, peripheral swelling/edema, and re-epithelialization). The average score of these parameters is shown in the graph. **(E)** Number of CFUs per swab, taken in between dressing changes or at the termination of the experiment. After the dressing changes, wound fluids were collected from the dressings followed by various assays for the control and *P. aeruginosa* wounds. Enzymatic activity of each collected fluid measured using **(F)** gelatine zymograms (representative experiments; U, uninfected; P, *P. aeruginosa* infected) or **(G)** azocasein. **(H)** IL-6 and **(I)** TNF-α levels in the wound fluids were analyzed using ELISA. Results are means ± SEM of 3 pigs per bacterial species, with in total 6 control wounds and 10–12 infected wounds. Values are significantly (*p < 0.05, **p < 0.005, ***p < 0.0005) different between groups at day 4 **(D)** or within the groups during time **(H,I)** as analyzed using an ordinary **(D)** or repeated measures **(H,I)** one-way ANOVA with Tukey’s multiple comparisons test. Solid lines, time-dependent differences within infected wounds; dashed lines, differences within uninfected wounds. Statistical comparisons between the groups for each day were not conducted **(E–I)**.

Despite extensive research on the virulence factors of *Pseudomonas aeruginosa*, the persistence of these bacteria in non-healing wounds remains a formidable challenge. *P. aeruginosa* is present in 50% of all venous leg ulcers and is uniquely correlated with delayed healing (33–35). The presence of *P. aeruginosa* in wound beds can manifest clinical and bacteriological signs of infection without the classical inflammatory responses (swelling, heat, redness), as illustrated in Figure 1A, indicating a failure of the immune system to recognize or combat these bacteria effectively. To enhance treatment strategies, it is imperative to deepen our understanding of the mechanisms by which *P. aeruginosa* modulates host responses in infected wounds. Drawing from this context, we hypothesized that *P. aeruginosa* actively supresses host inflammatory responses, rather than the attenuated inflammation being solely attributable to compromised host immunity, and we set out to identify the bacterial virulence factors responsible for this immunomodulatory effect.

Employing a translational porcine wound infection model, we successfully reproduced the observed lack of inflammatory responses. Furthermore, *in vitro* analyses using cell cultures and clinical isolates from venous ulcers and blood, as well as purified elastase, revealed that this protease degrades various pro-inflammatory cytokines and chemokines in the extracellular milieu, without affecting transcription factor activation or subsequent intracellular cytokine production.

## Materials and methods

### Ethics statement

This study was carried out in accordance with the recommendations of the Ethics Committee at Lund University, Lund, Sweden with written informed consent from all subjects in accordance with the Declaration of Helsinki. The protocols for the use of human blood (permit no. 657-2008), human wound materials and data (LU 708-01, LU 509-01) and pig models (M131-16) were approved by the Ethics Committee at Lund University.

### Proteins, proteases, and inhibitors

Carrier free recombinant cytokines were obtained from R&D systems (United Kingdom) and Genscript (United States). *P. aeruginosa* elastase (LasB) was isolated as described previously (36) with minor modifications. One unit of elastase is defined as the amount of enzyme capable of digesting 1 mg azocasein (Sigma-Aldrich, United States) per hour at 37°C in 80 μL reaction volume as determined with the azocasein assay described below. The MMP inhibitor GM6001 was obtained from Millipore (United States).

### Bacterial strains and growth conditions

*P. aeruginosa* clinical isolates were derived from patients with non-healing venous ulcers as previously described (37). Isolates from patient blood cultures were obtained from the Department of Medical Microbiology (Lund University) and had previously been species determined using MALDI-TOF mass spectrometry. *Staphylococcus aureus* ATCC 29213, *P. aeruginosa* strains PAO1 and PAOB1 [kindly provided by B. Iglewski (38)], and *P. aeruginosa* clinical isolates were grown in Todd-Hewitt medium at 37°C under vigorous shaking. For the growth curves, a small amount of medium was taken out of the culture at the indicated timepoints, and the absorbance (OD620 nm) was measured directly or after dilution when outside the measuring range of the instrument (followed by multiplication with the dilution factor).

To obtain conditioned medium (CM), bacteria from overnight cultures were grown for 24 h, centrifuged at 4,000 × g for 15 min and the culture media were sterilized through a 0.22 μm filter. For all experiments, a minimum of two independently generated batches of CM per isolate were used.

For the minipig studies, *S. aureus* ATCC 29213 and *P. aeruginosa* PAO1 in mid-logarithmic phase were washed and suspended in 10 mM Tris (pH 7.4) to 2 × 10^8^ CFU/mL.

### Biological materials

Wound fluids from patients with non-healing venous ulcers chronically colonized with *P. aeruginosa* were collected under a Tegaderm dressing for 2 h, as described previously (39). Sterile acute wound fluids, obtained from surgical drainages after mastectomy, were collected for 24 h, 24–48 h after surgery (40). Wound fluids were centrifuged and stored at –20°C. Human plasma was derived from fresh venous blood from healthy donors, collected in the presence of the anti-coagulant lepirudin (50 μg/mL). Plasma from Balb/c mice (female, 10–12 weeks; Scanbur, Denmark) injected intraperitoneally (i.p.) with 1 mg kg^–1^ *P. aeruginosa* LPS in 100 μL PBS was collected previously (32).

### Minipig model

To study *P. aeruginosa* wound infection *in vivo*, a minipig partial thickness wound model was set up. Six (three for each bacterial species) Göttingen minipigs (females) weighing 14–16 kg were acclimatized for 1 week prior to wounding. Approximately 24 h before surgery, hairs on the dorsum were clipped. The night before the start of the experiment, the minipigs were off-fed. On the day of wounding, the dorsum of the minipigs was cleaned with chlorhexidine soap (MEDI-SCRUB sponge, Rovers, The Netherlands) and lukewarm water, followed by shaving with a razor, disinfection with a chlorhexidine solution (4%) and drying with a sterile gauze. Under general anesthesia, 12 partial thickness wounds (750 μm deep), measuring 2.5 × 2.5 cm, were created on the back of the minipigs (6 on each side, between thoracic vertebrae 4–13) using an electric dermatome (Zimmer). A minimum distance of 4 cm was kept in between wounds. To establish an infection, *P. aeruginosa* and *S. aureus* bacterial suspensions were made in a neutral hydrogel (hydroxyethylcellulose; 10^7^ CFUs of *S. aureus* or *P. aeruginosa*/100 μL) and applied on the fresh wound surface. For the control wounds, 100 μL of gel without bacteria was applied. After 15 min, 500 μL of hydrogel was applied to the wounds and wounds were then covered with a primary foam dressing (Mepilex^®^ Transfer; Mölnlycke, Göteborg, Sweden). This primary dressing was then covered with a transparent breathable fixation dressing (Mepore^®^ Film; Mölnlycke, Göteborg, Sweden). For better fixation, dressings were secured with skin staples (SMI, Belgium). The entire wound area was then covered with two layers of sterile cotton gauze and secured with adhesive tape. Finally, a layer of flexible self-adhesive bandage (Vet Flex, Kruuse, Denmark) was used to support and protect the dressings underneath. All procedures were performed following strict aseptic techniques.

After recovery from anesthesia, minipigs were provided with water and feed and monitored for any discomfort. After 24 h, dressings were removed, and observations were recorded. Wounds were documented by imaging and clinical scoring. Swab samples were taken from the wound surface and used for bacterial analysis. Recovered primary dressings were used to collect wound fluids for further analysis. Before putting on new dressings, 500 μL of hydrogel was applied to both infected and control wounds. Dressings were changed once a day, and the experiment was terminated on the 4th day after infection. Finally, tissue samples from the wounds were collected for histological analysis.

### Clinical scoring of minipig wounds

Clinical scoring of minipig wounds was done for 6 separate parameters using the below scoring method. The average combined value of these parameters was calculated for each wound and used for comparison.

*Dressing stickiness:* 1 = not sticky, 2 = slightly sticky, 3 = moderately sticky, 4 = very sticky, 5 = hard to remove;

*Exudate amount:* 1 = none, dry wound, 2 = scant, moist wound but no observable exudate, 3 = small, 4 = moderate, 5 = large;

*Exudate type:* 1 = none, 2 = bloody, 3 = serosanguineous (thin, watery, pale red/pink), 4 = serous (thin, watery, clear), 5 = purulent (thin or thick, opaque or yellow);

*Necrotic tissue amount:* 1 = non-visible, 2 = < 25% of wound bed covered, 3 = 25–50% of wound bed covered, 4 = 50–75% of wound bed covered, 5 = > 75% of wound bed covered;

*Peripheral swelling/edema****:*** 1 = none, 2 = minimal, 3 = moderate swelling/edema, 4 = significant, 5 = extensive;

*Re-epithelization:* 1 = 100%, intact surface, 2 = 75–100%, 3 = 50–75%, 4 = 25–50%, 5 = < 25%.

### Wound fluid extraction

Wound dressings from the pig wounds were transferred to a 5 mL prechilled tube and kept on ice till extraction. To extract wound fluids, dressings were soaked in 500 μL of cold 10 mM Tris, pH 7.4, and centrifuged twice for 5 min (1,000 g, 4°C). Extracted wound fluids were aliquoted in prechilled Eppendorf tubes and stored in –80°C until further analysis.

### Bacterial analysis of wounds

Swabs collected from wounds were placed in 500 μL PBS. After vortexing for 30 s to release the bacteria, the samples were serially diluted, plated onto TH agar plates and incubated overnight at 37°C for CFU analysis.

### Histology

Harvested tissue samples from minipigs were fixed overnight in neutral buffered formalin and then stored in 70% ethanol. After serial dehydration, the tissues were embedded in paraffin blocks, sectioned and stained with hematoxylin and eosin (H&E). Samples were imaged with bright field microscopy (Axioplan2, Zeiss, Germany) using 10x and 40x magnifications.

### Enzymatic activity assay

Conditioned medium, porcine wound fluids and/or 10 mM Tris buffer (30 μL total volume) was incubated with 50 μL of 2% (w/v) azocasein in H_2_O for the indicated time points at 37°C. Next, 240 μL of a 10% (w/v) TCA solution was added to the sample to precipitate the proteins, followed by centrifugation at 8,000 × g for 5 min. Finally, 125 μL supernatant, containing cleaved off azo dye, was transferred to wells of a flat-bottom 96-wells plate containing 125 μL 1 M NaOH and the absorbance was measured at 450 nm.

### Zymograms

Gels were prepared consisting of a separation gel [0.1% (w/v) gelatine, 0.1% (w/v) SDS, 10% acrylamide in 375 mM Tris buffer (pH 8.8), 0.05% (v/v) TEMED and 0.05% (w/v) APS] and a stacking gel [0.1% (w/v) SDS, 4% acrylamide in 125 mM buffer (pH 6.8), 0.1% (v/v) TEMED, and 0.05% (w/v) APS]. Conditioned media were mixed with sample buffer [20% (v/v) glycerol, 5% (w/v) SDS, 0.03% (w/v) bromophenol blue, 0.4 M Tris-HCl pH 6.8] in a 1:1 ratio, transferred to the slots, and gels were run in electrophoresis buffer [25 mM Tris, 0.2 M glycine and 0.5% (w/v) SDS in H_2_O at pH 8.7] for 60 min at 150 V. Next, gels were washed in H_2_O, incubated for 1 h in 2.5% Triton X-100, washed again and placed in enzyme buffer (5 mM CaCl_2_, 1 μM ZnCl_2_, 200 mM NaCl and 50 mM Tris-HCl pH 7.5). After overnight incubation at 37°C while shaking (50 rpm), gels were washed and stained using Coomassie brilliant Blue. Enzymatic activity was observed after destaining the gels with a solution consisting of 10% EtOH and 14% acetic acid in H_2_O.

### SDS-gel electrophoresis

Carrier free recombinant cytokines were incubated with *P. aeruginosa* elastase or wound fluids for various time intervals at 37°C. Next, samples were denatured at 85°C for 5 min in 1x reducing SDS sample buffer followed by separation on Tricine mini gels in 1x Tricine SDS running buffer for 90 min at 120 V. Gels and buffers were derived from Novex^®^, Life Technologies, United States. Gels were stained with GelCode™ Blue Safe Protein Stain (ThermoScientific, United States) and patterns were visualized using a Gel Doc Imager (Bio-Rad Laboratories, United States).

### Western blotting

After SDS-gel electrophoresis, gels were transferred (25 V, 1.5 A for 11 min) to 0.2 μm PVDF membranes (Bio-Rad) using a Trans-Blot Turbo transfer system (Bio-Rad) according to manufacturer’s instructions. Subsequently, the membranes were rinsed in H_2_O, blocked (5% non-fat dry milk in PBS containing 0.05% Tween-20) for 60 min at RT while rotating, and incubated overnight at 2–8°C with polyclonal antibodies against TNF-α (rabbit anti-human, Invitrogen #p300A) or G-CSF (goat anti-human, R&D systems #AF-214-SP) while rotating. Next, the membranes were washed thrice in PBS supplemented with 0.05% Tween-20 and incubated with respectively swine anti-rabbit and rabbit anti-goat immunoglobulin—HRP conjugated antibodies (DAKO A/S, Denmark) for 60 min at RT while rotating. Finally, the membranes were washed, developed with SuperSignal West Pico Chemiluminescent Substrate (Thermo Scientific, Denmark) and visualized using a ChemiDoc XRS Imager (Bio-Rad).

### Whole blood assay

Fresh venous blood from healthy donors, collected in the presence of lepuridin (50 μg/mL), was mixed with 3 parts RPMI 1640—GlutaMAX™-I (Gibco^®^). Next, 1 mL of this mixture was transferred to each well of a 24-wells plate and stimulated with a range of various CMs or with *P. aeruginosa* LPS (Sigma Chemical Co., United States) in the absence and presence of purified elastase. After 18–22 h incubation at 37°C and 5% CO_2_, supernatants were collected and stored at –70°C.

### Isolation and stimulation of monocytes

PBMCs from healthy donors were isolated and stimulated as described previously (41). In short, buffy coats were subjected to Ficoll Amidotrizoate (ρ = 1.077 g/mL) density centrifugation at 700 × g for 20 min. Cells from the interphase were washed three times with PBS, and monocytes were purified using anti-CD14 coated Microbeads (Miltenyi Biotec GmbH, Germany). Approximately 1 × 10^6^ monocytes/mL of RPMI-1640 supplemented with 1x anti-anti and 10% heat-inactivated fetal bovine serum were transferred to wells of a 24-wells plate and incubated with a range of CM, in the presence or absence of the metalloprotease inhibitor GM6001, or stimulated with *P. aeruginosa* LPS in the absence or presence of purified elastase. After 18–22 h incubation at 37°C and 5% CO_2_, supernatants were collected and stored at –70°C.

### NF-κB and AP-1 activity measurement

THP1-Xblue™-CD14 reporter cells (InvivoGen, France) were cultured following manufacturer’s instructions. Cells were stimulated with 10% CM. After 18–22 h incubation at 37°C and 5% CO_2_, 20 μL of the supernatants were transferred to new 96-wells plates and 180 μL of QUANTI-Blue™ (InvivoGen) was added. Plates were incubated at 37°C and the levels of secreted embryonic alkaline phosphatase (SEAP), which is controlled by the activation of transcription factors NF-κB and AP-1, were measured after 1–2 h at an absorbance of 600 nm.

### Intracellular cytokine and chemokine measurement

Analysis was performed using a flow cytometer on fresh venous whole blood incubated as described above in the presence of 2 μM monensin. After 5 h incubation, erythrocytes were lysed using BD Pharm Lyse™ followed by 15 min incubation of the cells with conjugated mAbs directed against CD14 (#560180), CD3 (#563109) and CD20 (#563067) in staining buffer. Next, cells were fixed and permeabilized followed by 30 min incubation of the cells with conjugated mAbs directed against IL-12 (p40/p70, #565023), TNF-α (#557647), IL-6 (#554544), MCP-1 (#563496), and IFN-γ (#560704) in BD Horizon™ Brilliant Stain Buffer. All buffers, monensin and conjugated antibodies were derived from BD. Intracellular cytokine levels were measured on a FACSVerse (BD) and analyzed using FlowJo software (version 9.3.1). Median fluorescence intensities (MFI) of unstained samples were subtracted from the stained samples.

### Quantification of cellular phenotypes

Cellular morphologies were quantified using an automated microscopy-based High Content Analysis (HCA) platform. Briefly, THP-1 cells were seeded onto 96-well flat bottom plates at a density of 1.5 × 10^4^ cells/well in DMEM medium with or without a range of CM (in duplicate) and incubated at 37°C and 5% CO_2_ up to 48 h. Cells were stimulated with 100 ng/ml LPS or 100 nM PMA as controls. After incubation, cells attached to the plates were fixed with 3% formaldehyde in PBS for 10 min, whereas the non-attached cells were first transferred onto Poly-L-Lysine coated plates and incubated for 1 h, before fixation. Next, cells were fluorescently stained with Cell Mask™ and Hoechst (both from Molecular Probes^®^, Thermo Fisher Scientific Inc., United States). Plates were scanned (6 randomly selected fields/well at 20x magnification) using an automated microscope IN Cell Analyzer 2200 (GE Healthcare, Singapore). Images for whole well cell counting were acquired using 4x objective lens. Quantitative analysis of the acquired images was performed using IN Cell Investigator software (GE Healthcare, version 3.7.3) and the median values of the numerical data were converted into color-coded heatmaps for better visualization using GraphPad Prism. To obtain high resolution confocal images, cells were fixed with 3% formaldehyde for 24 h and stained with Alexa Fluor 647 Phalloidin (Molecular Probes^®^), FITC conjugated anti α-tubulin (Sigma) and Hoechst (Sigma). Confocal images were captured with a Zeiss LSM 800 with Airyscan, using a Plan-Apochromat 63x/1.4 oil immersion objective lens and 405 nm, 488 nm and 640 nm laser excitations. Confocal images were processed using Zen 2.1 lite imaging software (Carl Zeiss).

### Measurement of cytokine levels

Cytokine levels in human whole blood and monocyte culture samples were assessed using BioSource CytoSet™ (Invitrogen) and the Proteome Profiler™ Human Cytokine Array Panel A (R&D systems) following manufacturer’s instructions. The results of the Array were normalized, and the median values converted into color-coded heatmaps for better visualization using Graphpad Prism. Pig wound fluids collected from dressing material were analyzed using Porcine IL-6 and TNF-a DuoSet^®^ ELISA kits (R&D Systems, Minneapolis, United States) and mouse plasma using the BD Cytometric Bead Array Mouse Inflammation Kit (BD).

### Hemolysis assay

Fresh venous blood from healthy donors, collected in the presence of the anti-coagulant lepirudin (50 μg/mL), was centrifuged at 250 × g, and the pellet was washed 3 times with PBS. Next, the pellet was diluted 100 times, and 100 μL of this solution was transferred to each well of a 96-wells plate containing 100 μL of sample in PBS. Alternatively, 50 μL of blood was transferred directly to wells containing 150 μL of sample in RPMI. After 1 h incubation at 37°C and 5% CO_2_, the plate was centrifuged at 800 × g, 150 μL of each sample was transferred to a flat-bottom 96-wells plate and the absorbance at 450 nm was measured. Results are expressed as percentage of erythrocyte lysis compared to the positive control (2.5% Tween-20). Values below 10% are regarded as non-hemolytic.

### LDH assay

THP1-XBlue-CD14 and regular THP-1 cells were incubated with CM or purified elastase overnight. Next, LDH release in 50 μL cell culture media was measured using a lactic acid dehydrogenase based *in vitro* cytotoxicity assay kit (Pierce^®^, ThermoScientific) according to manufacturer’s instructions.

### Mitochondrial activity measurement

THP1-XBlue-CD14 cells were incubated with CM as above. Next, MTT [3-(4,5-dimethylthiazol-2-yl)-2,5 diphenyltetrazolium bromide; 5 mg/mL in PBS] was added to the wells to a final volume of 10% (v/v), followed by incubation at 37°C for 1–2 h. Finally, the supernatants were carefully removed, 100 μL of DMSO was added to the cells, and the reduction of MTT to formazan crystals was measured calorimetrically at 550 nm.

### Statistical analysis

All analyses were performed in GraphPad Prism. To calculate the differences between *in vitro* control samples and those incubated with CM or elastase, an unpaired *t*-test (when comparing two groups) or a repeated measures one-way ANOVA with Dunnett’s multiple comparisons tests (when comparing more than two groups) was performed, while a paired *t*-test or a repeated measures one-way ANOVA with Tukey’s multiple comparisons test was performed when calculating differences between the different samples at a given condition (concentration/cytokine/CM). *In vivo* samples were analyzed using an ordinary ANOVA (when comparing *P. aeruginosa* with *S. aureus*) or a repeated measures one-way ANOVA (when comparing the different days in the same group) with Tukey’s multiple comparisons test. The significances obtained with the *post-hoc* tests are indicated in the figures.

Correlation analysis was performed using the Pearson correlation coefficient. A *P*-value of < 0.05 was considered significant.

## Results

### Comparison of *P. aeruginosa* infections with Gram-positive infections in wounds

Infected wounds, such as those caused by Gram-positive bacteria, typically elicit pronounced inflammatory responses, characterized by erythema, edema, and elevated local temperature (42). However, clinical observations also show that these symptoms can be absent in wounds colonized or infected with the Gram-negative *Pseudomonas aeruginosa* (exemplified in Figure 1A). To investigate the *in vivo* reproducibility of these clinical observations, we established a porcine wound infection model using either *Pseudomonas aeruginosa* or the Gram-positive *Staphylococcus aureus.* For this purpose, we created 2.5 × 2.5 cm partial thickness wounds in Göttingen minipigs, infected them with *P. aeruginosa* PAO1 or with *S. aureus* ATCC 29213 and changed the dressings once a day for 2 subsequent days followed on day 4 by termination of the experiment as shown in Figure 1B. Wound appearance (Figure 1C) and clinical score (Figure 1D) at day 4 showed a slight increase in inflammation for *P. aeruginosa* infected wounds as compared to uninfected wounds, whereas infection with the Gram-positive *Staphylococcus aureus* resulted in wounds with clear signs of inflammation and necrosis, high cell infiltration and high clinical scores. Analysis of swabs, taken at each dressing change showed similar levels of colony forming units (CFUs) for the two strains [Figure 1E; additional data on *S. aureus* infections is reported in a separate study by Puthia et al (43)].

Interestingly, analyses of the wound fluids collected from each dressing showed increased numbers of proteases (each band corresponds to one protease) as well as protease activity in *P. aeruginosa* infected wounds as compared to uninfected wounds (Figure 1F). Further analysis of protease activity using an azocasein assay confirmed these results but showed no significant differences between the different days within each group. Notably, the infection-induced levels of pro-inflammatory cytokines IL-6 and TNF-α decreased time-dependently (Figures 1H,I; solid lines), whereas for uninfected wounds, the levels of TNF-α significantly increased over time (Figure 1H; dashed lines). Together, these proof of principle *in vivo* data are consistent with clinical observation that the presence of *P. aeruginosa* may result in non-healing wounds lacking the classical signs of infection and inflammation.

### Protease production by *P. aeruginosa* clinical isolates and effects on cytokines

**FIGURE 2:**
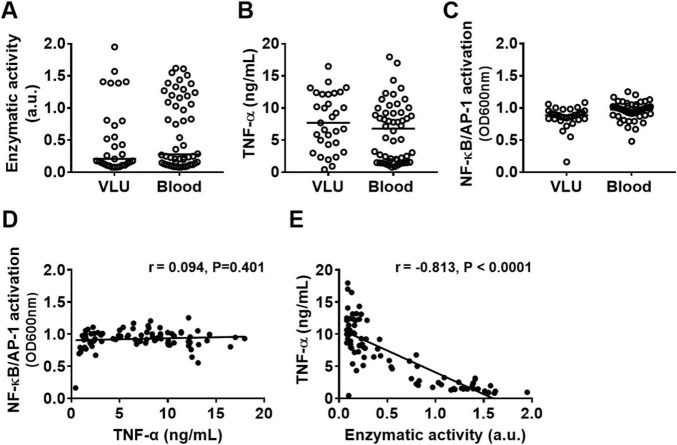
Protease production by *P. aeruginosa* clinical isolates and effects on TNF-α levels. Comparison of **(A)** enzymatic activity of 24 h CM from clinical *P. aeruginosa* isolates from bloodstream infections with those from venous leg ulcers, their ability to induce **(B)** TNF-α release in whole blood and **(C)** NF-κB/AP-1 activation in reporter THP1 cells after stimulation with 10% CM for 20 h. **(D)** The correlation between NF-κB/AP-1 activation in THP1 reporter cells and TNF levels in blood or **(E)** between enzymatic activity in CM and TNF levels in blood from all bacterial isolates combined was analyzed using the Pearson correlation coefficient (two-tailed).

Given the observed absence of inflammatory symptoms in the wounds, we investigated whether *Pseudomonas aeruginosa* clinical isolates from non-healing venous leg ulcers (VLU), that did not enter the bloodstream, differ from those from bloodstream infections, where inflammation is evident (e.g., fever), in their capacity to modulate cytokines and activate immune cells. For this purpose, we generated conditioned medium (CM) from 31 *P. aeruginosa* isolates from venous leg ulcers and 51 isolates from blood cultures and compared elastase activity, TNF-α levels after stimulating human whole blood (25%) and NF-κB/AP-1 activation by THP1-XBlue-CD14 reporter cells. As shown in Figures 2A–C, no significant differences were observed between the blood and VLU isolates, indicating that the ability to activate cells or produce proteases are not determining factors for the type of *P. aeruginosa* infection. While no correlation was found between the levels of cell activation (as measured by NF-κB/AP-1 activation) and TNF-α levels (Figure 2D), we did find a clear negative Pearson correlation coefficient (*r* = –0.813, *p* < 0.0001, slope –6.8 ± 0.5) between protease activity in CM and TNF-α levels found in incubated whole blood for the isolates (Figure 2E) suggesting that protease activity is a main factor for reduced cytokine levels.

Notably, a detailed overview of protease activity of the blood isolates depending on the severity of the infection (i.e., bacteremia, sepsis, septic shock, death), the origin of infection, and whether the patient was immunocompromised or not is shown in Supplementary Figure S1.

### Cytokine and chemokine levels are reduced in cell cultures stimulated with conditioned medium of elastase producing *P. aeruginosa*

**FIGURE 3:**
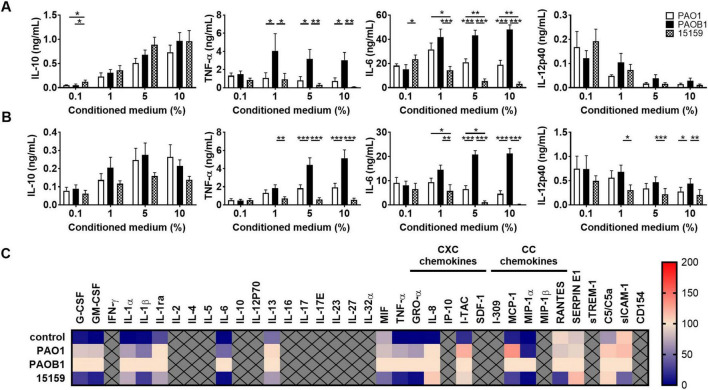
Elastase-containing conditioned medium reduces cytokine and chemokine levels. Human monocytes **(A)** or 25% human whole blood **(B)** were incubated for 18–22 h at 37°C with 0.1–10% conditioned medium of *P. aeruginosa* strains PAO1, PAOB1 or clinical isolate 15159 and supernatants were analyzed using ELISA. Results are means ± SEM of 6–9 **(A)** or 10–13 **(B)** experiments. Values are significantly (*p < 0.05, **p < 0.005, ***p < 0.0005) different as analyzed using a repeated measures one-way ANOVA with a Tukey’s multiple comparisons test. **(C)** Heatmap of normalized cytokine and chemokine (both CXC and CC) levels obtained with Proteome Profiler™ Arrays of whole blood stimulated with 10% CM or non-stimulated cells as a control (*n* = 4) (See also Supplementary Table S1).

As the above results suggested a role for bacterial proteases, and both alkaline protease (AprA) and the metalloprotease elastase B (LasB) can degrade casein (44) and TNF-α (25), we next investigated which of these enzymes in bacterial conditioned medium was mainly responsible for reduction of the cytokine and chemokine levels in whole blood. For this purpose, we used CM of lab strain PAO1 and the elastase B mutant strain PAOB1, both producing AprA [Supplementary Figure S2A for analysis on gelatine zymograms (44)], as well as the high protease producing clinical strain 15159 [which was isolated and first reported by Schmidtchen et al (37)]. As CM contains high levels of lipopolysaccharides (LPS) and other virulence factors that will activate cells, we did not add any additional stimuli. We found that CM of elastase producing strains PAO1 and 15159 (see Supplementary Figures S2A,B for enzymatic activity and growth curves of each strain) dose-dependently reduced TNF-α, and IL-6 levels in monocyte cultures (Figure 3A) and in 25% whole blood cultures (Figure 3B) as compared to the *lasB* mutant strain PAOB1. Notably, IL-12p40 levels decreased for all strains when increasing the amount of CM, although the elastase containing CMs significantly reduced the levels further in whole blood, indicating that besides degradation by elastase, other factors are involved too that regulate the level of this cytokine. Boiling of CM for 15 min or treatment with the metalloprotease inhibitor GM6001 abrogated the observed reduction in cytokine levels (Supplementary Figures S2C,D).

To further investigate the range of cytokines and chemokines that can be modulated by CM, whole blood samples were also analyzed using Proteome Profiler™ arrays (Figure 3C). We found that CM of elastase producing strain PAO1, as compared to the elastase mutant PAOB1, resulted in significantly decreased levels of the cytokines IL-1β, IL-6, the CXC chemokine GRO-α, and the CC chemokine MIP-1α (the means ± SEM and *P*-values are shown in Supplementary Table S1). Notably, the high elastase producing strain 15159 induced a further reduction in these levels and additionally decreased the levels of the cytokines G-CSF, GM-CSF, IL-1ra, IL-13, MIF, and TNF-α, the CC chemokine MCP-1, and sICAM-1. Levels produced by control, non-stimulated, cultures are shown for reference. Notably, the used amounts of CM were not hemolytic (Supplementary Figures S3A,B), although there was an increase in LDH release for all strains, with 10% CM reaching LPS-induced levels of LDH release (Supplementary Figure S3C), as well as a dose-dependent reduction in mitochondrial activity (Supplementary Figure S3D).

### *P. aeruginosa* elastase reduces cytokine and chemokine levels

As the above differences between strains PAO1 and 15159 could be explained either by the higher levels of elastase in CM of 15159, or by differences in the levels of other virulence factors or proteases without elastolytic activity, we investigated the effect of purified elastase (see Supplementary Figure S4 for HPLC profile) on the cytokine levels in cell cultures stimulated with *Pseudomonas*-derived lipopolysaccharides (LPS), using 100 ng/mL which has been reported in infected leg ulcers (45).

**FIGURE 4:**
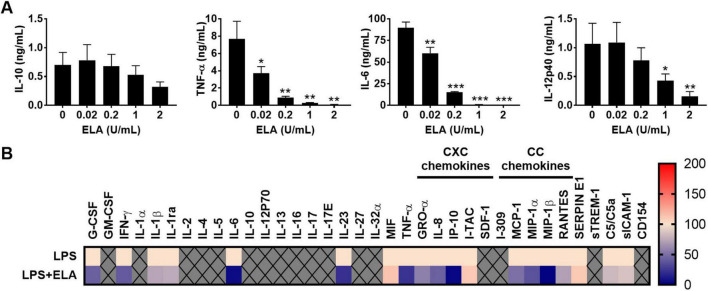
*P. aeruginosa* elastase reduces cytokine and chemokine levels. **(A)** Human monocytes were incubated for 18–22 h at 37°C with LPS (100 ng/mL) and a range of *P. aeruginosa* elastase (ELA). Results are means ± SEM of 3 experiments. Values are significantly (*p < 0.05, **p < 0.005, ***p < 0.0005) different compared to the control as analyzed using a repeated measures one-way ANOVA with a Dunnett’s multiple comparisons test. **(B)** Heatmap of normalized cytokine and chemokine (CXC and CC sub-families) levels obtained with Proteome Profiler™ Arrays of 25% human whole blood cultures stimulated with LPS (100 ng/mL) and elastase (2 units/mL; *n* = 4) (see also Supplementary Table S2).

In monocyte-cultures, elastase induced a dose-dependent reduction of the levels of the proinflammatory cytokines TNF-α, IL-12p40 and IL-6, whereas IL-10 levels were not significantly affected (Figure 4A). The corresponding levels found for control, non-stimulated cells were: 0 pg/ml of IL-10, 10.7 ± 8.7 pg/mL of TNF-α, 4.3 ± 2.3 pg/mL of IL-12p40 and 7.0 ± 3.6 pg/mL of IL-6. To further investigate the range of cytokines and chemokines that can be modulated by elastase, samples from 25% whole blood cultures incubated with LPS and elastase (2 U/mL) were analyzed using Proteome Profiler™ Arrays (Figure 4B). The results show significantly reduced levels of cytokines (G-CSF, IFN-γ, IL-1ra, IL-6, IL-23, and TNF-α) and chemokines (IL-8, IP-10, MCP-1, and MIP-1β) in the presence of elastase as compared to LPS alone (Supplementary Table S2). The other tested markers were not significantly altered or not detected.

Notably, while most cytokines exhibited similar trends in response to purified elastase and CM from PAO1 and 15159, the results for MIF, IL-8, and MCP-1 deviated from the CM results. This may be attributed to varying levels of elastase activity or the influence of other components present in elastase-containing CM, which may interfere with the effects of elastase alone.

### *P. aeruginosa* elastase is produced by many clinical strains and does not affect cell morphology or activation

**FIGURE 5:**
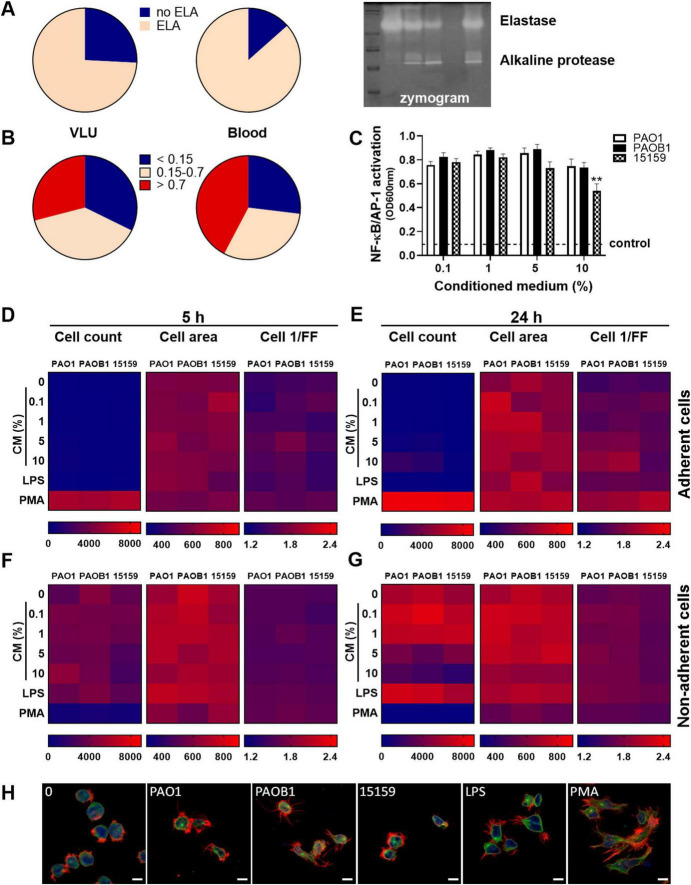
*P. aeruginosa* elastase is produced by many clinical strains and does not affect cell characteristics. Conditioned medium of the clinical isolates from venous leg ulcers and from blood were analyzed for elastase production (yes/no) and protease activity (0–2 arbitrary units) using respectively zymograms **(A)** and azocasein assays **(B)**. A representative example of a zymogram is shown **(A**, right panel**)**. **(C)** NF-κB/AP-1 activation by various amounts of CM of the strains PAO1, PAOB1, and 15159. Values are significantly (**p < 0.005) different from the 0.1% CM value as analyzed using a repeated measures one-way ANOVA with a Dunnett’s multiple comparisons test. **(D–G)** Heatmaps of high content analysis of THP-1 cells stimulated with a range of CM, LPS (100 ng/mL) or PMA (100 nM) for 5 h **(D,F)** and 24 h **(E,G)**; **(D,E)** adherent cells, **(F,G)** non-adherent cells. Medians are shown of 3 independent experiments performed in duplicate, with 2 independently generated batches of CM for each strain per experiment. **(H)** Representative confocal microscopy images of control cells or cells incubated with 10% CM for 24 h. Cells were fixed and stained with Hoechst (DNA; blue), Alexa Fluor 647 Phalloidin (F-actin; red) and FITC anti α-tubulin (green). Scale bar: 10 μm.

To investigate the extent of elastase production among *P. aeruginosa* isolates, we tested CM of the clinical isolates on gelatine zymograms. We found that 74% of all venous leg ulcer isolates (*n* = 31) and 87% of the blood isolates (*n* = 52) produced elastase under the used culture conditions (Figure 5A). Next, we measured the level of protease activity using azocasein and found that respectively 39 and 31% of the VLU and blood strains showed medium (OD between 0.15 and 0.7) protease activity, 29 and 42% high (OD > 0.7) activity, whereas the remaining 32 and 27% showed low or no activity (Figure 5B).

To further investigate cell activation, the dose- and time-dependent effects of CM of the strains PAO1, PAOB1, and 15159 were analyzed for NF-κB/AP-1 activity in THP1 reporter cells (Figure 5C) and high content analysis (HCA) using THP-1 cells (Figures 5D–G). All strains induced similar levels of NF-κB/AP-1 activation, besides 10% CM of strain 15159, which resulted in a small but significant decreased level as compared to the 0.1% level.

Using HCA, we observed that the control phorbol 12-myristate 13-acetate (PMA) induced significant adherence of the cells at 5 h (Figure 5D) and 24 h (Figure 5E). In agreement, clear morphological alterations, due to plate adherence, could be observed for PMA-stimulated cells (Figure 5H). None of the bacterial CMs induced substantial cell adherence (Figures 5D–G) and no significant differences were observed in cell area and cell roundness (cell 1/FF) for the different stimuli and concentrations for both the adherent and non-adherent cells at 5 h (Figures 5D,F) and 24 h (Figures 5E,G). Of note, the observed decrease in non-adherent cells after 24 h incubation in the presence of 10% CM is likely due to toxicity. As shown in Supplementary Figures S3C,F, increasing amounts of CM resulted in increased LDH release in both cell types, although there was no significant difference in LDH release between the three strains. Furthermore, we observed a significant dose-dependent reduction in mitochondrial activity for all CMs, while 10% CM of 15159 was significantly lower than 10% CM of PAO1. As the number of cells used for the HCA assay is 20 times less than used for the other cell assays, the toxic effects are expected to be more pronounced.

Collectively, the results demonstrate that most clinical strains of *P. aeruginosa* tested in this study produce elastase, which does not seem to affect cell morphology or activation by CM, as no differences were observed between PAO1 and PAOB1.

### *P. aeruginosa* elastase does not inhibit intracellular cytokine production

**FIGURE 6:**
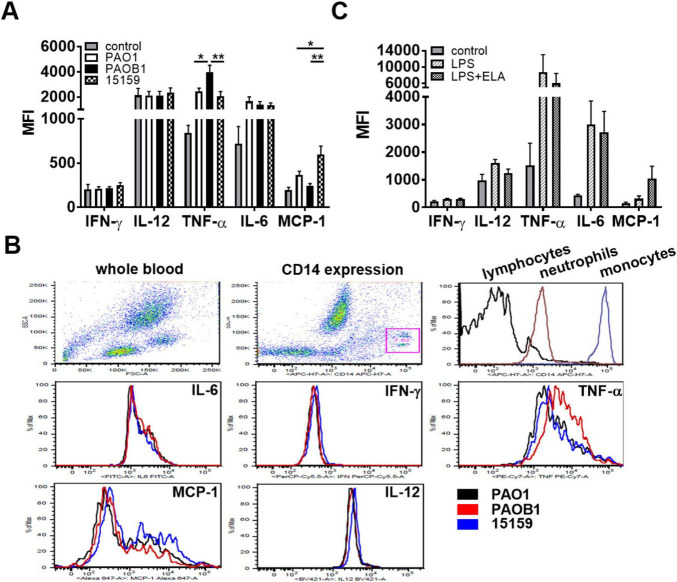
*P. aeruginosa* elastase does not inhibit intracellular cytokine production. Human whole blood supplemented with 2 μM monensin was incubated for 5 h with **(A,B)** 10% CM or **(C)** LPS (1 μg/mL) with or without elastase (2 units/mL) and intracellular cytokine levels were measured using FACS. Results are the average median fluorescent intensities (MFI) ± SEM of 3 **(C)** and 4 **(A**, two batches of CM per donor**)** blood donors. Values are significantly (*p < 0.05, **p < 0.005) different as analyzed using a repeated measures one-way ANOVA with a Tukey’s multiple comparisons test. **(B)** Representative examples of the FACS plots: dot plots of forward-sideward scatter and gating of monocytes based on CD14 expression, and histograms of CD14 expression of the three cell types and of the five tested cytokines in monocytes.

As elastase reduced cytokine levels but did not interfere with cell activation, we next investigated whether cytokine levels were modulated intracellularly. Using whole blood, we did not find significantly altered levels of intracellular cytokines in either lymphocytes or neutrophils in response to 5 h incubation with 10% CM or LPS as compared to non-stimulated cells (data not shown), whereas in monocytes several cytokines were upregulated (Figure 6A). In these latter cells, we found comparable induction of intracellular IL-6 production for the different strains, whereas TNF-α production was significantly increased for PAOB1 (Figures 6A,B); IFN-γ and IL-12p40/70 were not significantly upregulated by the CMs as compared to the control samples. Additionally, purified elastase did not significantly reduce the production of IL-6, IFN-γ, or TNF-α in response to LPS stimulation (Figure 6C). Interestingly, MCP-1 production was significantly upregulated in the presence of CM containing elastase and a clear trend was observed for purified elastase (Figures 6A,C), although with the latter, high donor variations prevented obtaining significance. Taken together, these results indicate that the reduced cytokine and chemokine levels observed in whole blood and monocyte cultures are not caused by inhibited production.

### *P. aeruginosa* elastase degrades cytokines and chemokines released to the extracellular space

**FIGURE 7:**
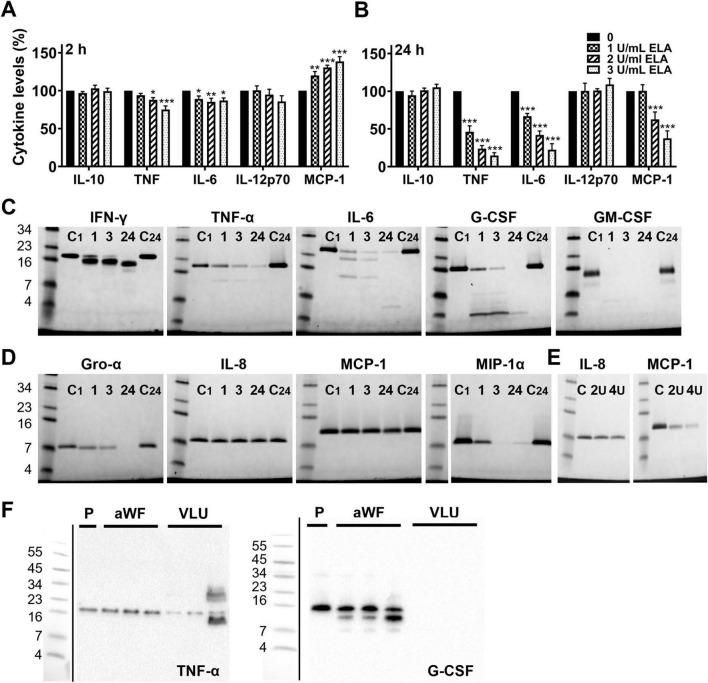
*P. aeruginosa* elastase degrades cytokines and chemokines. Plasma derived from mice injected with LPS was incubated with a range of elastase for 2 h **(A)** and 24 h **(B)**, and cytokine levels were analyzed using FACS. Results are normalized values of 4 independent experiments and are significantly (*p < 0.05, **p < 0.005, ***p < 0.0005) different from the control as analyzed using a repeated measures one-way ANOVA with a Dunnett’s multiple comparisons test. **(C)** Recombinant cytokines and **(D)** chemokines were incubated with elastase (1 U/mL) for 1, 3 or 24 h and degradation was visualized using SDS-PAGE. C1, control incubated for 1 h; C24, control incubated for 24 h. **(E)** Effect of 2 and 4 units elastase/mL on IL-8 and MCP-1 after 24 h incubation. **(F)** To investigate whether cytokines and chemokines are degraded in infected wounds, wound fluids from non-infected acute wounds (aWF) or *P. aeruginosa* colonized/infected venous leg ulcers (VLU) were spiked with recombinant human TNF-α or G-CSF and incubated for 24 h at 37°C, followed by analysis using SDS-PAGE and western blotting (P, plasma used as a control; representative blots are shown).

Next, we investigated whether elastase degrades cytokines extracellularly. As we previously showed that purified elastase resulted in significantly reduced levels of IL-6, TNF, and MCP-1 in mice that were injected with CM of PAOB1 (32), we used plasma from the control mice from this same study that were injected with LPS, and incubated it with increasing concentrations of purified elastase for 2 h or 24 h at 37° C followed by FACS analysis. In agreement with Figures 3C, 4B, the results showed a dose- and time-dependent decrease in the levels of IL-6, TNF and MCP-1 (Figures 7A,B), whereas IFN-γ was below the detection limit. IL-10 and IL-12p70 levels were not affected by elastase. Interestingly, reduced levels of MCP- 1 were found at 24 h, while at 2 h the levels were increased.

To confirm that the reduced cytokine levels were due to degradation, we incubated a selection of recombinant cytokines (Figure 7C) and chemokines (Figure 7D) with 1 U/mL of purified elastase and analyzed the samples using SDS-PAGE. In agreement with the results above, elastase time-dependently degraded the cytokines IFN-γ, TNF-α, IL-6, G-CSF and GM-CSF, and the chemokines Gro-α and MIP-1α. While IL-8 and MCP-1 were not affected by 1 U/mL of elastase, 2 and 4 U/mL showed degradation of MCP-1 as well, whereas IL-8 remained visually unaffected (Figure 7E). Finally, we investigated whether cytokines and chemokines can be degraded by patient wound fluids. For this purpose, we incubated recombinant TNF-α or G-CSF for 24 h with wound fluids from sterile acute wounds (aWF) or from *P. aeruginosa* colonized/infected venous leg ulcers (VLU) and analyzed cytokine degradation using SDS-PAGE and western blotting. The results showed less intact TNF-α and no detectable G-CSF (Figure 7F) after incubation with fluids from colonized/infected wounds as compared to non-infected acute wounds or cytokine in plasma (P) as a control, indicating that cytokines indeed can be degraded in wounds colonized/infected with *P. aeruginosa*. Notably, endogenous TNF-α and G-CSF were not detected in these wound fluids, whereas acute wound fluids induced cleavage of G-CSF.

## Discussion

*Pseudomonas aeruginosa* is a Gram-negative opportunistic pathogen renowned for its immune evasive capabilities. This study was initiated based on clinical observations that venous wounds colonized/infected with *P. aeruginosa* often lack overt signs of inflammation, allowing this bacterium to persist in wounds for prolonged periods (35), leading to impaired wound healing (33, 34, 46). Since patients with non-healing wounds are typically elderly with venous insufficiencies, the absence of infection signs might be attributed solely to a compromised immune response. To investigate this, we developed a porcine wound infection model using immunocompetent pigs. We observed significant inflammation in wounds infected with *Staphylococcus aureus*, whereas the inflammatory response to *P. aeruginosa* was only moderate. Interestingly, *P. aeruginosa* infections led to increased protease activity and a time-dependent decrease in cytokine levels, while control uninfected wounds exhibited a time-dependent increase in cytokine levels and low protease activity. Collectively, these findings indicate that impaired host responses are not the primary factor for the observed lack of inflammation.

As our results suggested a role for proteases in reducing cytokine levels, and as both AprA and elastase have been shown to cleave a large variety of host proteins, we selected PAO1, the *lasB* mutant PAOB1 (which does produce AprA) and a range of clinical isolates to elucidate the underlying mechanism. In cell and blood cultures, we found that cytokine and chemokine levels were reduced by purified *P. aeruginosa* elastase, as well as conditioned medium of elastase-producing strains, whereas there was no correlation between cytokine levels and activation of transcription factors NF-κB and AP-1, which indicated that the observed effects were not caused by modulation of signal transduction. Indeed, intracellular cytokine levels of selected cytokines and chemokines in monocytes were not affected to such an extent that it could explain the above observations. Instead, we found that elastase degraded the recombinant cytokines G-CSF, GM-CSF, IFN-γ, IL-6, and TNF-α and chemokines Gro-α, MIP-1α, and MCP-1 time-dependently. In our whole blood assay, we additionally observed significantly reduced levels of IL-1ra, IL-12p40, IL-23, IL-8, IP-10, and MIP-1β in the presence of purified elastase, although for these we cannot exclude indirect effects of elastase, e.g., by activation of host proenzymes, such as several matrix metalloproteases (47–49). Collectively, our findings corroborate previous research demonstrating that elastase B degrades the proinflammatory cytokines IFN-γ (25), TNF-α (25), and IL-6 (50) and align with our prior studies indicating that elastase reduces pro-inflammatory cytokine levels in experimental murine models *in vivo* (32). Notably, although elastase degraded MCP-1 as reported previously (26), we did find increased intracellular MCP-1 levels in the presence of elastase containing CM and purified elastase. Differences in elastase levels between CM of PAO1 and 15159 might therefore explain the observed enhanced levels in whole blood by the former, while the levels were decreased by the higher elastase levels in the latter. Although *P. aeruginosa* nitrite reductase can induce MCP-1 production in human pulmonary type II epithelial-like cells (51), to our knowledge there is no previous evidence suggesting that elastase can enhance this process or exert a similar effect in monocytes. The reason for the increased MCP-1 levels remains to be investigated. Notably, although extracellular MCP-1 levels were decreased when incubating LPS-stimulated plasma with elastase for 24 h, incubation for 2 h showed increased MCP-1 levels. As MCP-1 can’t be produced in cell-free plasma, these results may be explained by a lack of exposure of the antibody recognition site on the MCP-1 molecules in the control samples, whereas elastase increased this exposure.

From a substrate perspective, the differences in cleavage efficiency of the different proteins by elastase can be explained by substrate specificity. At the N-terminal position, elastase cleaves peptide bonds at the non-polar amino acids leucine (L), valine (V), isoleucine (I), phenylalanine (F) and tryptophan (W), as well as the non-charged polar tyrosine (Y) (27, 52). At the C-terminal amino acid cleavage side, elastase does not have a clear specificity, although an increase of glycine (G), proline (P), threonine (T), serine (S), lysine (K), and glutamate (E) was observed (27). However, analysis of specific cleavage sites for the different cytokines and chemokines was outside the scope of this study.

Importantly, it should be noted that a substantial part of our clinical isolates (∼25%) did not produce elastase under standard culturing conditions in Todd Hewitt (TH) broth. These data should however be treated with caution, as the used *in vitro* conditions do not reflect the complex physiological microenvironment bacteria encounter during infection. Moreover, *P. aeruginosa* produces a range of other virulence factors that will modulate host inflammatory responses in the absence of elastase. Therefore, future experiments should be directed to examining the protease expression *in situ* in *P. aeruginosa* infected wounds.

Of further clinical relevance is the observation that *P. aeruginosa* isolates from non-healing venous ulcers and blood cultures exhibited no significant differences in proinflammatory cell responses or protease activity *in vitro*. This is noteworthy as acute inflammation is prominent feature in *P. aeruginosa* bacteremia. Clearly, other virulence factors may differ between the two groups. However, it has been shown that, except for chemotactic motility, there are few infection-type-specific genetic pathways required for fitness in one infection vs. the other (53). Instead, in burn wounds it has been shown that arginine levels may serve as a crucial environmental cue for *P. aeruginosa* to switch between a sessile, biofilm-forming lifestyle and a motile one (54, 55). Additionally, human albumin has been reported to inhibit quorum sensing and subsequent production of virulence factors, including elastase, *in vitro* (56). This finding has potential clinical implications for wound healing and infection development (57). Collectively, these insights highlight the complexity of the wound environment, where both host and bacterial factors jointly influence the outcome of infection.

From a translational and physiological perspective, the link between *P. aeruginosa* infection, proteolysis and cytokine degradation is further substantiated by our finding that fluids from wounds colonized/infected with elastase-producing *P. aeruginosa* are indeed degrading cytokines whereas fluids from non-infected wounds do not. Moreover, the selected endogenous cytokines could not be detected in the infected wound fluids, supporting our conclusion that cytokines are degraded in the highly proteolytic wound environment.

Clearly, with this information as background, future clinical prospective studies are needed to correlate overall bacterial protease production, including elastase and others, to wound status and specific host characteristics, such as inflammation markers and levels of various proteins including albumin, arginine, and cytokines during *P. aeruginosa* infection.

## Limitations of the study

Considering the *in vivo* porcine wound infection model, we created single species infections of short duration, whereas in wounds, infections are typically polymicrobial, leading to microbial interactions that could alter the results, and are of longer duration. Also, although we observed increased protease activity together with time-dependent decreased IL-6 and TNF-α levels, we do not have solid proof that this is due to degradation, as decreased production of cytokines and chemokines cannot be excluded. Nevertheless, the results obtained with our model resemble our clinical observations and are compatible with the *in vitro* results.

## Data Availability

The raw data supporting the conclusions of this article will be made available by the authors, without undue reservation.

## References

[1] Emerson J Rosenfeld M McNamara S Ramsey B Gibson R. *Pseudomonas aeruginosa* and other predictors of mortality and morbidity in young children with cystic fibrosis. *Pediatr Pulmonol.* (2002) 34:91–100. 10.1002/ppul.10127 12112774

[2] Lyczak J Cannon C Pier G. Lung infections associated with cystic fibrosis. *Clin Microbiol Rev.* (2002) 15:194–222. 10.1128/CMR.15.2.194-222.2002 11932230 PMC118069

[3] Vincent J Sakr Y Sprung C Ranieri V Reinhart K Gerlach H Sepsis in European intensive care units: Results of the SOAP study. *Crit Care Med.* (2006) 34:344–53. 10.1097/01.ccm.0000194725.48928.3a 16424713

[4] Crousilles A Maunders E Bartlett S Fan C Ukor E Abdelhamid Y Which microbial factors really are important in *Pseudomonas aeruginosa* infections? *Future Microbiol.* (2015) 10:1825–36. 10.2217/fmb.15.100 26515254

[5] Klockgether J Tümmler B. Recent advances in understanding *Pseudomonas aeruginosa* as a pathogen. *F1000Res.* (2017) 6:1261. 10.12688/f1000research.10506.1 28794863 PMC5538032

[6] Galdino A Branquinha M Santos A Viganor L. *Pseudomonas aeruginosa* and Its arsenal of proteases: Weapons to battle the host. In: <snm>Chakraborti S</gnm>, <snm>Dhalla N</gnm> editors. *Pathophysiological Aspects of Proteases.* Singapore: Springer Singapore (2017). p. 381–97.

[7] Sainz-Mejías M Jurado-Martín I McClean S. Understanding *Pseudomonas aeruginosa*-host interactions: The ongoing quest for an efficacious vaccine. *Cells.* (2020) 9:2617. 10.3390/cells9122617 33291484 PMC7762141

[8] Liao C Huang X Wang Q Yao D Lu W. Virulence factors of *Pseudomonas Aeruginosa* and antivirulence strategies to combat its drug resistance. *Front Cell Infect Microbiol.* (2022) 12:926758. 10.3389/fcimb.2022.926758 35873152 PMC9299443

[9] Qin S Xiao W Zhou C Pu Q Deng X Lan L *Pseudomonas aeruginosa*: Pathogenesis, virulence factors, antibiotic resistance, interaction with host, technology advances and emerging therapeutics. *Signal Transduct Target Ther.* (2022) 7:199. 10.1038/s41392-022-01056-1 35752612 PMC9233671

[10] Kida Y Higashimoto Y Inoue H Shimizu T Kuwano K. A novel secreted protease from *Pseudomonas aeruginosa* activates NF-kappaB through protease-activated receptors. *Cell Microbiol.* (2008) 10:1491–504. 10.1111/j.1462-5822.2008.01142.x 18331590

[11] Patzer J Nielsen H Kharazmi A. *Pseudomonas aeruginosa* exotoxin A primes human monocyte oxidative burst response in vitro. *Microb Pathog.* (1989) 7:147–52. 10.1016/0882-4010(89)90033-8 2512464

[12] Pedersen S Kharazmi A Espersen F Høiby N. *Pseudomonas aeruginosa* alginate in cystic fibrosis sputum and the inflammatory response. *Infect Immun.* (1990) 58:3363–8. 10.1128/iai.58.10.3363-3368.1990 2401567 PMC313661

[13] Otterlei M Sundan A Skjåk-Braek G Ryan L Smidsrød O Espevik T. Similar mechanisms of action of defined polysaccharides and lipopolysaccharides: Characterization of binding and tumor necrosis factor alpha induction. *Infect Immun.* (1993) 61:1917–25. 10.1128/iai.61.5.1917-1925.1993 8478081 PMC280784

[14] Jaeger K Kharazmi A Høiby N. Extracellular lipase of *Pseudomonas aeruginosa*: Biochemical characterization and effect on human neutrophil and monocyte function in vitro. *Microb Pathog.* (1991) 10:173–82. 10.1016/0882-4010(91)90052-c 1910141

[15] Vareechon C Zmina S Karmakar M Pearlman E Rietsch A. *Pseudomonas aeruginosa* effector ExoS inhibits ROS production in human neutrophils. *Cell Host Microbe.* (2017) 21:611–8.e5. 10.1016/j.chom.2017.04.001 28494242 PMC5478421

[16] Sanchez Klose F Dahlstrand Rudin A Bergqvist L Scheffler J Jönsson K Islander U The *Pseudomonas aeruginosa* lectin LecB modulates intracellular reactive oxygen species production in human neutrophils. *Eur J Immunol.* (2024) 54:e2350623. 10.1002/eji.202350623 37972111

[17] Lindsay S Oates A Bourdillon K. The detrimental impact of extracellular bacterial proteases on wound healing. *Int Wound J.* (2017) 14:1237–47. 10.1111/iwj.12790 28745010 PMC7949928

[18] Nagano T Hao J Nakamura M Kumagai N Abe M Nakazawa T Stimulatory effect of pseudomonal elastase on collagen degradation by cultured keratocytes. *Invest Ophthalmol Vis Sci.* (2001) 42:1247–53. 11328735

[19] Peters J Park S Darzins A Freck L Saulnier J Wallach J Further studies on *Pseudomonas aeruginosa* LasA: Analysis of specificity. *Mol Microbiol.* (1992) 6:1155–62. 10.1111/j.1365-2958.1992.tb01554.x 1588815

[20] Heck L Morihara K Abrahamson D. Degradation of soluble laminin and depletion of tissue-associated basement membrane laminin by *Pseudomonas aeruginosa* elastase and alkaline protease. *Infect Immun.* (1986) 54:149–53. 10.1128/iai.54.1.149-153.1986 3093382 PMC260129

[21] Heck L Morihara K McRae W Miller E. Specific cleavage of human type III and IV collagens by *Pseudomonas aeruginosa* elastase. *Infect Immun.* (1986) 51:115–8. 10.1128/iai.51.1.115-118.1986 3079727 PMC261073

[22] Engel L Hill J Caballero A Green L O’Callaghan R. Protease IV, a unique extracellular protease and virulence factor from *Pseudomonas aeruginosa*. *J Biol Chem.* (1998) 273:16792–7. 10.1074/jbc.273.27.16792 9642237

[23] Horvat R Parmely M. *Pseudomonas aeruginosa* alkaline protease degrades human gamma interferon and inhibits its bioactivity. *Infect Immun.* (1988) 56:2925–32. 10.1128/iai.56.11.2925-2932.1988 3139565 PMC259672

[24] Theander T Kharazmi A Pedersen B Christensen L Tvede N Poulsen L Inhibition of human lymphocyte proliferation and cleavage of interleukin-2 by *Pseudomonas aeruginosa* proteases. *Infect Immun.* (1988) 56:1673–7. 10.1128/iai.56.7.1673-1677.1988 3133317 PMC259461

[25] Parmely M Gale A Clabaugh M Horvat R Zhou W. Proteolytic inactivation of cytokines by *Pseudomonas aeruginosa*. *Infect Immun.* (1990) 58:3009–14. 10.1128/iai.58.9.3009-3014.1990 2117578 PMC313603

[26] Leidal K Munson K Johnson M Denning G. Metalloproteases from *Pseudomonas aeruginosa* degrade human RANTES, MCP-1, and ENA-78. *J Interferon Cytokine Res.* (2003) 23:307–18. 10.1089/107999003766628151 12859857

[27] Cai J Nielsen M Kalogeropoulos K. Auf dem Keller U, van der Plas MJA. Peptidomic analysis of endogenous and bacterial protease activity in human plasma and wound fluids. *iScience.* (2024) 27:109005. 10.1016/j.isci.2024.109005 38333691 PMC10850760

[28] Laarman A Bardoel B Ruyken M Fernie J Milder F van Strijp J *Pseudomonas aeruginosa* alkaline protease blocks complement activation via the classical and lectin pathways. *J Immunol.* (2012) 188:386–93. 10.4049/jimmunol.1102162 22131330

[29] Heck L Alarcon P Kulhavy R Morihara K Russell M Mestecky J. Degradation of IgA proteins by *Pseudomonas aeruginosa* elastase. *J Immunol.* (1990) 144:2253–7. 10.4049/jimmunol.144.6.2253 2107256

[30] Schmidtchen A Frick I Andersson E Tapper H Björck L. Proteinases of common pathogenic bacteria degrade and inactivate the antibacterial peptide LL-37. *Mol Microbiol.* (2002) 46:157–68. 10.1046/j.1365-2958.2002.03146.x 12366839

[31] Hartman E Forsberg F Kjellström S Petrlova J Luo C Scott A Peptide clustering enhances large-scale analyses and reveals proteolytic signatures in mass spectrometry data. *Nat Commun.* (2024) 15:7128. 10.1038/s41467-024-51589-y 39164298 PMC11336174

[32] van der Plas MJA Bhongir R Kjellström S Siller H Kasetty G Mörgelin M *Pseudomonas aeruginosa* elastase cleaves a C-terminal peptide from human thrombin that inhibits host inflammatory responses. *Nat Commun.* (2016) 7:11567. 10.1038/ncomms11567 27181065 PMC4873665

[33] Gjødsbøl K Christensen J Karlsmark T Jørgensen B Klein B Krogfelt K. Multiple bacterial species reside in chronic wounds: A longitudinal study. *Int Wound J.* (2006) 3:225–31. 10.1111/j.1742-481X.2006.00159.x 16984578 PMC7951738

[34] Bjarnsholt T Kirketerp-Møller K Jensen PØ Madsen KG Phipps R Krogfelt K Why chronic wounds will not heal: A novel hypothesis. *Wound Repair Regen.* (2008) 16:2–10. 10.1111/j.1524-475X.2007.00283.x 18211573

[35] Renner R Sticherling M Rüger R Simon J. Persistence of bacteria like *Pseudomonas aeruginosa* in non-healing venous ulcers. *Eur J Dermatol.* (2012) 22:751–7. 10.1684/ejd.2012.1865 23220150

[36] Morihara K Tsuzuki H Oka T Inoue H Ebata M. *Pseudomonas aeruginosa* elastase. isolation, crystallization, and preliminary characterization. *J Biol Chem.* (1965) 240:3295–304. 10.1016/S0021-9258(18)97217-0 14321366

[37] Schmidtchen A Holst E Tapper H Björck L. Elastase-producing *Pseudomonas aeruginosa* degrade plasma proteins and extracellular products of human skin and fibroblasts, and inhibit fibroblast growth. *Microb Pathog.* (2003) 34:47–55. 10.1016/s0882-4010(02)00197-3 12620384

[38] Toder D Gambello M Iglewski B. *Pseudomonas aeruginosa* LasA: A second elastase under the transcriptional control of lasR. *Mol Microbiol.* (1991) 5:2003–10. 10.1111/j.1365-2958.1991.tb00822.x 1766376

[39] Schmidtchen A. Degradation of antiproteinases, complement and fibronectin in chronic leg ulcers. *Acta Derm Venereol.* (2000) 80:179–84. 10.1080/000155500750042925 10954207

[40] Lundqvist K Herwald H Sonesson A Schmidtchen A. Heparin binding protein is increased in chronic leg ulcer fluid and released from granulocytes by secreted products of *Pseudomonas aeruginosa*. *Thromb Haemost.* (2004) 92:281–7. 10.1160/TH03-12-0732 15269823

[41] van der Plas MJA Baldry M van Dissel J Jukema G Nibbering P. Maggot secretions suppress pro-inflammatory responses of human monocytes through elevation of cyclic AMP. *Diabetologia.* (2009) 52:1962–70. 10.1007/s00125-009-1432-6 19575178 PMC2723663

[42] Stulberg D Penrod M Blatny R. Common bacterial skin infections. *Am Fam Physician.* (2002) 66:119–24. 12126026

[43] Puthia M Butrym M Petrlova J Strömdahl A Andersson MÅ Kjellström S A dual-action peptide-containing hydrogel targets wound infection and inflammation. *Sci Transl Med.* (2020) 12:eaax6601. 10.1126/scitranslmed.aax6601 31894104

[44] Caballero A Moreau J Engel L Marquart M Hill J O’Callaghan R. *Pseudomonas aeruginosa* protease IV enzyme assays and comparison to other *Pseudomonas* proteases. *Anal Biochem.* (2001) 290:330–7. 10.1006/abio.2001.4999 11237336

[45] Trøstrup H Holstein P Christophersen L Jørgensen B Karlsmark T Høiby N S100A8/A9 is an important host defence mediator in neuropathic foot ulcers in patients with type 2 diabetes mellitus. *Arch Dermatol Res.* (2016) 308:347–55. 10.1007/s00403-016-1646-7 27084691

[46] Ruffin M Brochiero E. Repair process impairment by *Pseudomonas aeruginosa* in epithelial tissues: Major features and potential therapeutic avenues. *Front Cell Infect Microbiol.* (2019) 9:182. 10.3389/fcimb.2019.00182 31214514 PMC6554286

[47] Okamoto T Akaike T Suga M Tanase S Horie H Miyajima S Activation of human matrix metalloproteinases by various bacterial proteinases. *J Biol Chem.* (1997) 272:6059–66. 10.1074/jbc.272.9.6059 9038230

[48] Maeda H Okamoto T Akaike T. Human matrix metalloprotease activation by insults of bacterial infection involving proteases and free radicals. *Biol Chem.* (1998) 379:193–200. 10.1515/bchm.1998.379.2.193 9524071

[49] de Bentzmann S Polette M Zahm J Hinnrasky J Kileztky C Bajolet O *Pseudomonas aeruginosa* virulence factors delay airway epithelial wound repair by altering the actin cytoskeleton and inducing overactivation of epithelial matrix metalloproteinase-2. *Lab Invest.* (2000) 80:209–19. 10.1038/labinvest.3780024 10701690

[50] Saint-Criq V Villeret B Bastaert F Kheir S Hatton A Cazes A *Pseudomonas aeruginosa* LasB protease impairs innate immunity in mice and humans by targeting a lung epithelial cystic fibrosis transmembrane regulator-IL-6-antimicrobial-repair pathway. *Thorax.* (2018) 73:49–61. 10.1136/thoraxjnl-2017-210298 28790180 PMC5738602

[51] Sar B Oishi K Wada A Hirayama T Matsushima K Nagatake T. Induction of monocyte chemoattractant protein-1 (MCP-1) production by *Pseudomonas* nitrite reductase in human pulmonary type II epithelial-like cells. *Microb Pathog.* (2000) 28:17–23. 10.1006/mpat.1999.0320 10623560

[52] Rawlings N Waller M Barrett A Bateman A. MEROPS: The database of proteolytic enzymes, their substrates and inhibitors. *Nucleic Acids Res.* (2014) 42:D503–9. 10.1093/nar/gkt953 24157837 PMC3964991

[53] Turner K Everett J Trivedi U Rumbaugh K Whiteley M. Requirements for *Pseudomonas aeruginosa* acute burn and chronic surgical wound infection. *PLoS Genet.* (2014) 10:e1004518. 10.1371/journal.pgen.1004518 25057820 PMC4109851

[54] Bernier S Ha D Khan W Merritt J O’Toole G. Modulation of *Pseudomonas aeruginosa* surface-associated group behaviors by individual amino acids through c-di-GMP signaling. *Res Microbiol.* (2011) 162:680–8. 10.1016/j.resmic.2011.04.014 21554951 PMC3716369

[55] Everett J Turner K Cai Q Gordon V Whiteley M Rumbaugh K. Arginine Is a Critical Substrate for the Pathogenesis of *Pseudomonas aeruginosa* in Burn Wound Infections. *mBio.* (2017) 8:e2160–2116. 10.1128/mBio.02160-16 28292986 PMC5350470

[56] Smith A Rice A Sutton B Gabrilska R Wessel A Whiteley M Albumin inhibits *Pseudomonas aeruginosa* quorum sensing and alters polymicrobial interactions. *Infect Immun.* (2017) 85:e116–7. 10.1128/IAI.00116-17 28630071 PMC5563583

[57] Legendre C Debure C Meaume S Lok C Golmard J Senet P. Impact of protein deficiency on venous ulcer healing. *J Vasc Surg.* (2008) 48:688–93. 10.1016/j.jvs.2008.04.012 18579333

