# Supplementary material for: Pseudomonas aeruginosa elastase down-regulates host inflammatory responses by degrading cytokines and chemokines: a non-healing wound perspective
Source: Front Med (Lausanne). 2025 Jun 24;12:1585252. doi: 10.3389/fmed.2025.1585252 (PMC12234559; doi:10.3389/fmed.2025.1585252)
Supplement: Supplementary file 1 [file Table_1.docx]

**Supplemental table 1**: Statistical analysis of profiler array results of 25% whole blood incubated with 10% bacterial conditioned medium.

The means are given in percentages as compared to PAOB1, which was set to 100%. P- values were calculated using an unpaired t-test. No value means not detected.

|  | **Non-stimulated cells** | | |  | **PAO1** | | | |  | | **15159** | | | |
| --- | --- | --- | --- | --- | --- | --- | --- | --- | --- | --- | --- | --- | --- | --- |
|  | **Mean ± SEM** |  | **P-value** |  | **Mean ± SEM** |  | **P-value** |  | | **Mean ± SEM** | |  | **P-value** |  |
| G-CSF | 9.6 ± 5.8 |  | <0.0005 |  | 84.1 ± 11.3 |  | 0.2086 |  | | 25.2 ± 4.2 | |  | <0.0005 |  |
| GM-CSF | 4.2 ± 3.8 |  | <0.0005 |  | 82.3 ± 8.4 |  | 0.0789 |  | | 13.5 ± 3.9 | |  | <0.0005 |  |
| IFN-γ |  |  |  |  |  |  |  |  | |  | |  |  |  |
| IL-1α |  |  |  |  | 69.8 ± 15.0 |  | 0.1817 |  | | 75.0 ± 13.8 | |  | 0.2117 |  |
| IL-1β | 2.7 ± 1.8 |  | <0.0005 |  | 58.2 ± 8.6 |  | 0.0028 |  | | 28.5 ± 8.8 | |  | 0.0002 |  |
| IL-1ra | 28.5 ± 11.5 |  | 0.0007 |  | 124.2 ± 35.9 |  | 0.5254 |  | | 65.8 ± 7.6 | |  | 0.0042 |  |
| IL-2 |  |  |  |  |  |  |  |  | |  | |  |  |  |
| IL-4 |  |  |  |  |  |  |  |  | |  | |  |  |  |
| IL-5 |  |  |  |  |  |  |  |  | |  | |  |  |  |
| IL-6 | 0.7 ± 0.3 |  | <0.0005 |  | 49.5 ± 13.1 |  | 0.0085 |  | | 4.8 ± 2.0 | |  | <0.0005 |  |
| IL-10 |  |  |  |  |  |  |  |  | |  | |  |  |  |
| IL-12P70 |  |  |  |  |  |  |  |  | |  | |  |  |  |
| IL-13 | 48.8 ± 24.5 |  | 0.0676 |  | 106.0 ± 4.2 |  | 0.2325 |  | | 72.3 ± 6.1 | |  | 0.0102 |  |
| IL-16 |  |  |  |  |  |  |  |  | |  | |  |  |  |
| IL-17 |  |  |  |  |  |  |  |  | |  | |  |  |  |
| IL-17E |  |  |  |  |  |  |  |  | |  | |  |  |  |
| IL-23 |  |  |  |  |  |  |  |  | |  | |  |  |  |
| IL-27 |  |  |  |  |  |  |  |  | |  | |  |  |  |
| IL-32α |  |  |  |  |  |  |  |  | |  | |  |  |  |
| MIF | 105.4 ± 40.9 |  | 0.8816 |  | 72.3 ± 13.7 |  | 0.0899 |  | | 55.8 ± 12.2 | |  | 0.01105 |  |
| TNF-α | 0.6 ± 0.6 |  | <0.0005 |  | 80.2 ± 14.7 |  | 0.1682 |  | | 13.8 ± 3.5 | |  | <0.0005 |  |
| GRO-α | 0.7 ± 0.5 |  | <0.0005 |  | 55.9 ± 14.2 |  | 0.0210 |  | | 5.8 ± 1.7 | |  | <0.0005 |  |
| IL-8 | 2.5 ± 1.5 |  | <0.0005 |  | 97.4 ± 26.2 |  | 0.9252 |  | | 105.4 ± 11.9 | |  | 0.6660 |  |
| IP-10 |  |  |  |  |  |  |  |  | |  | |  |  |  |
| I-TAC | 37.1 ± 27.8 |  | 0.0425 |  | 116.2 ± 11.4 |  | 0.2057 |  | | 98.5 ±14.8 | |  | 0.9226 |  |
| SDF-1 |  |  |  |  |  |  |  |  | |  | |  |  |  |
| I-309 |  |  |  |  |  |  |  |  | |  | |  |  |  |
| MCP-1 | 29.6 ± 23.0 |  | 0.0146 |  | 199.1 ± 75.6 |  | 0.2379 |  | | 26.8 ± 9.7 | |  | <0.0005 |  |
| MIP-1α | 2.1 ± 2.1 |  | <0.0005 |  | 16.8 ± 6.1 |  | <0.0005 |  | | 5.0 ± 2.6 | |  | <0.0005 |  |
| MIP-1β |  |  |  |  |  |  |  |  | |  | |  |  |  |
| RANTES | 262.9 ± 206.0 |  | 0.3878 |  | 76.0 ± 23.1 |  | 0.3402 |  | | 42.1 ± 25.2 | |  | 0.0612 |  |
| SERPIN E1 | 76.6 ± 35.6 |  | 0.4673 |  | 82.3 ± 22.6 |  | 0.4637 |  | | 105.5 ± 35.4 | |  | 0.8816 |  |
| sTREM-1 |  |  |  |  |  |  |  |  | |  | |  |  |  |
| C5/C5a | 71.1 ± 19.3 |  | 0.1340 |  | 111.8 ± 17.4 |  | 0.5231 |  | | 111.5 ± 13.2 | |  | 0.4165 |  |
| sICAM-1 | 110.7 ± 13.1 |  | 0.3736 |  | 111.6 ± 8.2 |  | 0.2068 |  | | 40.4 ± 16.0 | |  | 0.0098 |  |
