# Supplementary material for: Pseudomonas aeruginosa elastase down-regulates host inflammatory responses by degrading cytokines and chemokines: a non-healing wound perspective
Source: Front Med (Lausanne). 2025 Jun 24;12:1585252. doi: 10.3389/fmed.2025.1585252 (PMC12234559; doi:10.3389/fmed.2025.1585252)
Supplement: Supplementary file 2 [file Table_2.docx]

**Supplemental table 2**: Statistical analysis of profiler array results of 25% whole blood stimulated with 100 ng/ml of LPS in the presence of 2 units/ml elastase.

The means are given in percentages as compared to LPS alone, which was set to 100%. P- values were calculated using an unpaired t-test. No value means not detected.

|  |  | **Mean ± SEM** |  | **P-value** |
| --- | --- | --- | --- | --- |
| G-CSF |  | 42.4 ± 19.0 |  | 0.038582 |
| GM-CSF |  |  |  |  |
| IFN-γ |  | 39.7 ± 0.1 |  | <0.000001 |
| IL-1α |  |  |  |  |
| IL-1β |  | 73.0 ± 13.1 |  | 0.084581 |
| IL-1ra |  | 75.3 ± 2.6 |  | 0.000069 |
| IL-2 |  |  |  |  |
| IL-4 |  |  |  |  |
| IL-5 |  |  |  |  |
| IL-6 |  | 5.2 ± 1.6 |  | <0.000001 |
| IL-10 |  |  |  |  |
| IL-12P70 |  |  |  |  |
| IL-13 |  |  |  |  |
| IL-16 |  |  |  |  |
| IL-17 |  |  |  |  |
| IL-17E |  |  |  |  |
| IL-23 |  | 21.3 ± 1.7 |  | <0.000001 |
| IL-27 |  |  |  |  |
| IL-32α |  |  |  |  |
| MIF |  | 113.2 ± 34.1 |  | 0.713480 |
| TNF-α |  | 22.6 ± 12.8 |  | 0.000926 |
| GRO-α |  | 56.5 ± 28.0 |  | 0.170701 |
| IL-8 |  | 43.0 ± 12.6 |  | 0.004005 |
| IP-10 |  | 3.4 ± 3.4 |  | <0.000001 |
| I-TAC |  | 110.7 ± 31.0 |  | 0.747550 |
| SDF-1 |  |  |  |  |
| I-309 |  |  |  |  |
| MCP-1 |  | 49.5 ± 17.1 |  | 0.025370 |
| MIP-1α |  | 36.4 ± 35.0 |  | 0.118310 |
| MIP-1β |  | 0.7 ± 0.7 |  | <0.000001 |
| RANTES |  | 70.6 ± 33.1 |  | 0.408141 |
| SERPIN E1 |  | 110.6 ± 12.4 |  | 0.425841 |
| sTREM-1 |  |  |  |  |
| C5/C5a |  | 81.4 ± 23.7 |  | 0.461401 |
| sICAM-1 |  | 85.7 ± 15.9 |  | 0.404256 |
