# Supplementary material for: Pseudomonas aeruginosa elastase down-regulates host inflammatory responses by degrading cytokines and chemokines: a non-healing wound perspective
Source: Front Med (Lausanne). 2025 Jun 24;12:1585252. doi: 10.3389/fmed.2025.1585252 (PMC12234559; doi:10.3389/fmed.2025.1585252)
Supplement: Supplementary file 3 [file Data_Sheet_1.docx]

**Supplementary Figures 1-4**

**
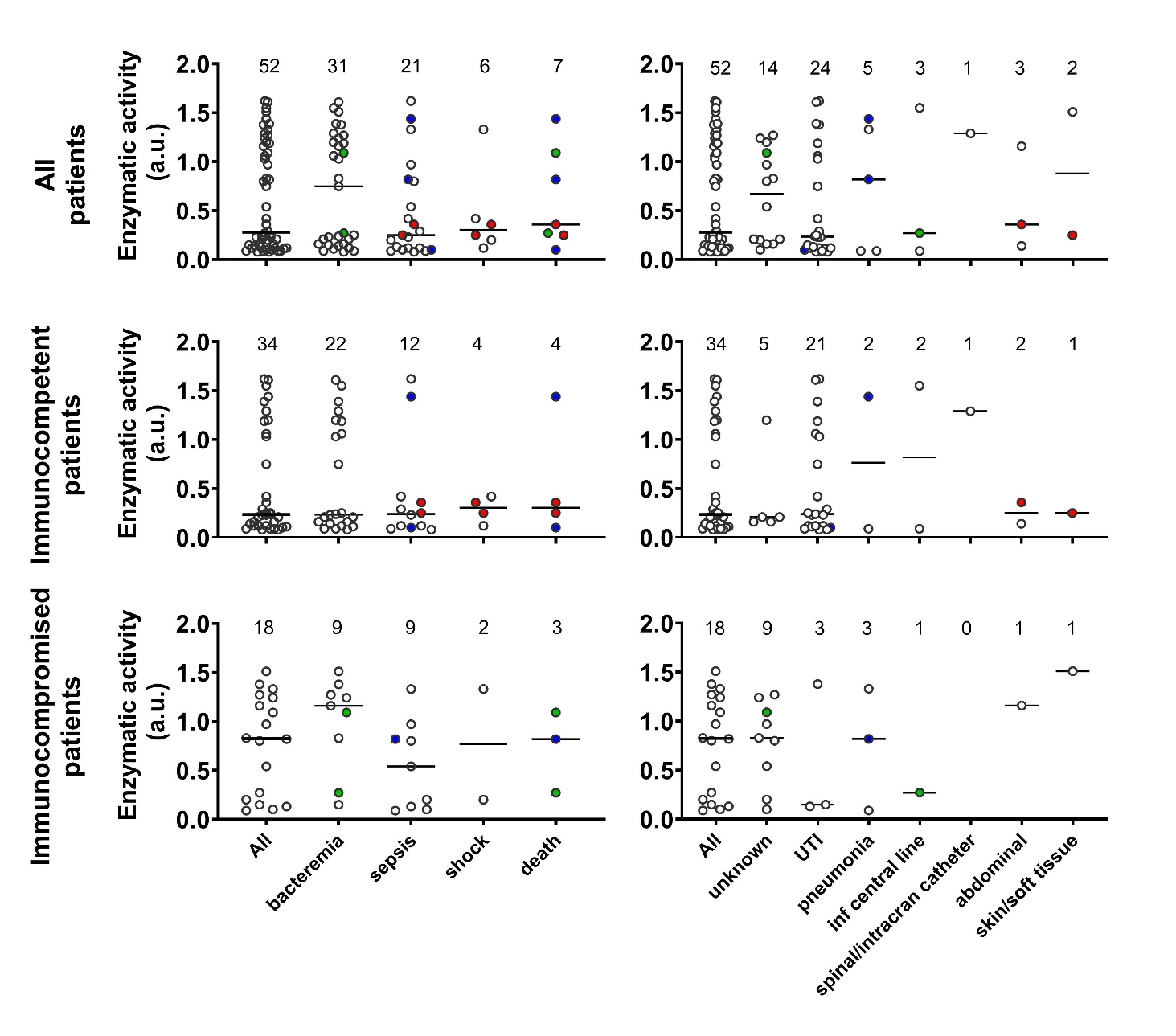
Supplementary Figure 1. Origin and disease severity of the blood isolates**

Enzymatic activity of conditioned medium derived from a total of 52 *P. aeruginosa* isolates, of which 34 originated from immunocompetent patients and 18 from immunocompromised individuals, was measured using azocasein as a substrate. Patients were classified into bacteremia or sepsis categories, with all cases of septic shock falling under the latter. Among the seven patients who succumbed, two were diagnosed with bacteremia (indicated by green dots), three with sepsis (indicated by blue dots), and the remaining three with septic shock (indicated by red dots). Additionally, classification was performed on the origin of the infection: urinary tract infection (UTI), pneumonia, infected central line, catheter-related spinal/intracranial, abdominal and skin/soft tissue infections.

**Supplementary Figure 2. Proteolytic activity by the *P. aeruginosa* strains**

(A) A representative zymogram showing elastase (ELA) and alkaline protease (AprA) activity by CM of strains PAO1, 15159 and PAOB1. (B) Azocasein (50 µl) was incubated for 2 h with CM (25 µL) at 37 °C followed by protein precipitation and centrifugation. Supernatants were transferred to 96-wells plates contain 1 M NaOH (1:1 ratio) and the absorbance was measured at 450 nm. Results, expressed as arbitrary units (a.u.), are means ± SEM of 3 independently generated batches of CM. Values are significantly (***p<0.0005) different as analysed using an ordinary one-way ANOVA with a Tukey’s multiple comparisons test. (C) Growth curves of the three strains in TH medium (n=4). The absorbance was either measured directly (values below 1.7) or after dilution of the samples followed by multiplication with the dilution factor (values above 1.7). (D) Human whole blood (25% in RPMI) was incubated for 20 – 24 h at 37 °C with 10% regular or boiled (15 min) CM of *P. aeruginosa* strains PAO1, PAOB1 or clinical isolate 15159. (E) Human monocytes were incubated for 20-24 h with 10% CM in the absence or presence of 10 µM GM6001. Supernatants were analysed using ELISA and results are means ± SEM of 6 (D) or 4-6 (E) experiments. Values are significantly (*p<0.05, **p<0.005, ***p<0.0005) different as analysed using a paired t-test.

** Supplementary Figure 3. Cell viability in the presence of *P. aeruginosa* CM or elastase**

Haemolysis of erythrocytes by CM present in 25% (A) or 0.5% (B) whole blood. Below the dotted line, samples are considered non-haemolytic. (C) CM-induced LDH-release and (D) metabolic activity in reporter THP1-XBlue-CD14 cells. (E) LDH-release by THP1-XBlue-CD14 cells in the presence of various amounts of elastase (ELA). F) CM-induced LDH-release in regular THP-1 cell cultures. LPS (100 ng/mL) and unstimulated controls were added for comparison (C, F). Results are means ± SEM of 8-9 (A, C, D, F), 11-12 (B) and 4 (E) experiments. Values are significantly (*p<0.05) different as analysed using a repeated measures one-way ANOVA with a Tukey’s multiple comparisons test.

Elastase

**Supplementary Figure 4. HPLC profile of purified elastase**

Purified elastase was injected into a HPLC system (PerkinElmer Series 200; PerkinElmer, USA) equipped with a Vydac 218TP C18 (5 μm, 4.6 mm × 250 mm) reverse phase column (Grace Discovery Sciences, USA). The mobile phases consisted of (A) 0.05% trifluroacetic acid in H_2_O and (B) 0.05% trifluroacetic acid in acetonitrile. A 70 min gradient from 5 to 95% B was applied at a flow rate of 1 ml min^−1^. The absorbance was monitored at 214 nm.
